# Spontaneous Splenic Rupture in a Patient With Recent Use of Performance-Enhancing Compounds: A Case Report and Literature Review

**DOI:** 10.7759/cureus.106106

**Published:** 2026-03-30

**Authors:** Kumail Jaffry, Jainam Shah, Niyaz Naqash

**Affiliations:** 1 Department of General Surgery and Acute Surgery Unit, Monash Health, Melbourne, AUS; 2 Department of Surgery, Monash University, Melbourne, AUS

**Keywords:** atraumatic splenic rupture, sarms, selective androgen receptor modulators, splenectomy, spontaneous splenic rupture

## Abstract

Atraumatic splenic rupture (ASR) is a rare but life-threatening surgical emergency that most commonly occurs in pathologically abnormal spleens. We present a unique case of ASR in a middle-aged man with recent use of performance-enhancing compounds, managed with both embolization and surgery. This case highlights an unusual potential contributing risk factor and underscores the importance of prompt diagnosis and definitive management of ASR.

A 54-year-old male presented to the emergency department with acute left upper quadrant abdominal pain and vomiting. Notably, he had recently been using MK-677 (ibutamoren), a growth hormone secretagogue, and RAD-140 (testolone), a selective androgen receptor modulator (SARM), for bodybuilding purposes. The patient denied any history of trauma. Computed tomography revealed a large perisplenic hematoma consistent with splenic rupture. Following initial resuscitation, emergent splenic artery embolization was performed to control hemorrhage. This intervention stabilized the patient transiently; however, he ultimately required an emergency laparotomy with total splenectomy. Histopathological examination demonstrated large areas of splenic infarction, with features raising the possibility of an underlying vascular malformation.

These findings underscore the importance of recognising ASR in patients with acute abdominal pain and vascular risk factors. We explore the potential and speculative role of performance-enhancing compounds in precipitating splenic complications, drawing parallels to the established association between traditional anabolic steroids and peliosis - blood-filled vascular cavities that predispose to rupture. While RAD-140 and MK-677 have not been previously linked to splenic pathology, their pharmacological effects on androgen receptor signalling and IGF-1 pathways warrant consideration as possible contributing factors, particularly in the context of a suspected pre-existing vascular malformation. Early recognition, aggressive resuscitation, and timely surgical intervention remain critical, as delayed diagnosis carries significant mortality.

## Introduction

Atraumatic splenic rupture (ASR) is an uncommon but potentially fatal condition, accounting for approximately 0.1% to 0.5% of all splenic injuries. However, some retrospective analyses suggest the incidence may reach 3% among all splenic ruptures [1,2]. The condition exhibits a male predominance with a sex ratio of approximately 2:1 and carries an estimated mortality rate between 12% and 20% [3]. Approximately one-third of patients present with signs of hemodynamic shock, and in about 8% of cases, the diagnosis is only established at autopsy [4,5]. Without a clear history of trauma, ASR is frequently overlooked even when classic features such as left upper quadrant pain, peritonism, and hemodynamic instability are present [6].

In the majority of cases, splenic rupture occurs in the context of underlying splenic pathology, with truly spontaneous rupture in a normal spleen being exceedingly rare. A comprehensive systematic review identified hematological malignancies (16.4%), such as acute myeloid leukemia and non-Hodgkin lymphoma, as the most common etiology, followed by viral infections (14.8%), including infectious mononucleosis and cytomegalovirus, and localized inflammatory processes (10.9%), such as pancreatitis [7]. Certain medications, particularly anticoagulants, have also been implicated, although the evidence remains limited [8]. Idiopathic rupture occurs in fewer than 10% of patients, and the underlying mechanism remains poorly understood.

Vascular anomalies of the spleen, including hemangiomas, hamartomas, and peliosis, represent a rare but recognized subset of ASR risk factors. Of particular relevance to this case is the established association between traditional anabolic steroid use and peliosis of the liver and spleen, an acquired vascular condition characterized by blood-filled sinusoidal cavities that can precipitate spontaneous rupture. Other drugs previously linked to hepatic or splenic vascular lesions include danazol and thiopurines, highlighting that exogenous hormonal or immunomodulatory agents represent an important pharmacological consideration in unexplained visceral hemorrhage [8].

Our patient's recent use of MK-677, a growth hormone secretagogue that elevates insulin-like growth factor-1 (IGF-1) levels via ghrelin receptor agonism, and RAD-140, a selective androgen receptor modulator (SARM), raises important questions about whether these newer performance-enhancing compounds might carry similar risks to traditional anabolic steroids. SARMs, while marketed as safer alternatives to conventional anabolic-androgenic steroids, are associated with a range of adverse effects, including suppression of the hypothalamic-pituitary-gonadal axis, hepatotoxicity, dyslipidemia, and emerging cardiovascular and prothrombotic risk [references]. MK-677, though not an SARM, similarly carries risks including insulin resistance, fluid retention, and sustained elevation of IGF-1 - a potent anabolic and mitogenic mediator with recognized effects on vascular biology. To our knowledge, there are no published reports directly linking SARMs or growth hormone secretagogues to splenic injury. This case, therefore, contributes novel insight by suggesting a temporal association, the clinical significance of which remains uncertain, and highlights the importance of obtaining a thorough history regarding over-the-counter bodybuilding supplements in patients presenting with unexplained visceral hemorrhage.

## Case presentation

A 54-year-old male presented to the emergency department with acute-onset, generalized abdominal pain localized predominantly to the left upper quadrant, associated with multiple episodes of vomiting. He reported recent use of two orally active anabolic compounds for weightlifting purposes in the four weeks: MK-677 (ibutamoren), a growth hormone secretagogue (ghrelin receptor agonist), and RAD-140 (testolone), a selective androgen receptor modulator. He had also been recovering from a dry cough presumed secondary to influenza in the preceding weeks. He denied any recent fevers, trauma, or history of abdominal surgery. There was no known hepatic or splenic pathology. His past medical history was significant only for anxiety and depression, managed with diazepam 5 mg, mirtazapine 45 mg, and olanzapine 20 mg nocte. He was an active smoker and consumed alcohol occasionally.

**Figure 1:**
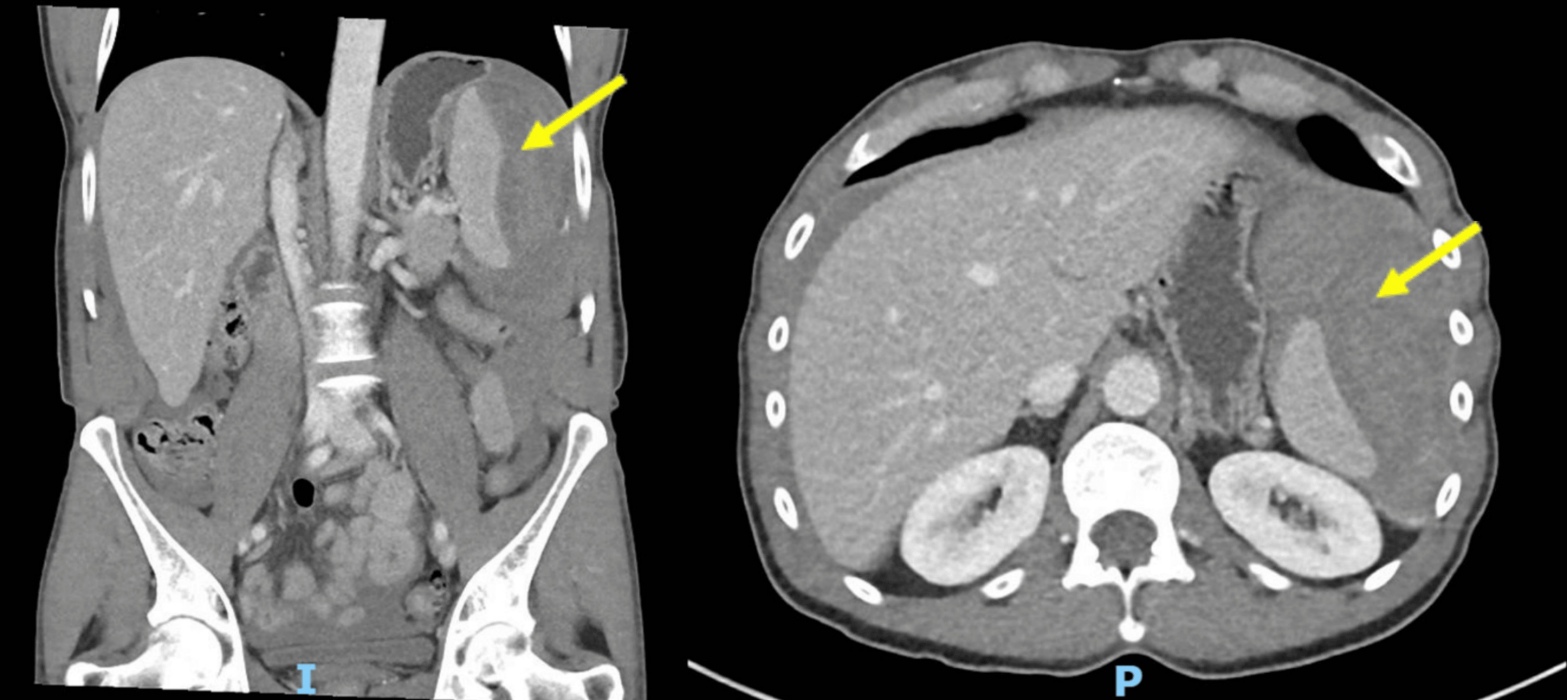
CT abdomen and pelvis demonstrating a large perisplenic hematoma (yellow arrow) consistent with splenic rupture. Coronal section on the left; axial section on the right. CT, computed tomography

On initial examination, the patient was hemodynamically stable with marked localized peritonism in the left hypochondrium. Laboratory investigations revealed leucocytosis at 17.2 × 10⁹/L, elevated C-reactive protein (CRP) of 12 mg/L, respiratory acidosis, and raised lactate of 2.7 mmol/L. His hemoglobin and liver function tests were within normal limits at presentation (Tables 1-2). Contrast-enhanced computed tomography (CT) of the abdomen and pelvis demonstrated a large perisplenic hematoma consistent with splenic rupture, which was subsequently confirmed on CT angiography, without evidence of active arterial extravasation (Figure 1).

**Table 1:** Pertinent laboratory results from admission (0 hrs) to splenic embolization (13 hrs). INR, international normalized ratio; CRP, C-reactive protein; APTT, activated partial thromboplastin time; WCC, white cell count; H, high; L, low

Timeframe	+0 hrs	+4 hrs	+7 hrs	+9 hrs	+11 hrs	+13 hrs
pH (7.30-7.43)	7.30	7.28 (L)	7.25 (L)	7.17 (L)	7.16 (L)	7.27 (L)
pCO2 (mmHg) (38-58)	58	61 (H)	60 (H)	60 (H)	63 (H)	49 (H)
Bicarbonate (22-32)	28	28	26	22	22	22
Lactate (0.5-2.0)	2.7 (H)	4.0 (H)	6.2 (H)	8.0 (H)	8.2 (H)	4.6 (H)
Hb (g/L) (130-180)	152	138	-	107 (L)	-	84 (L)
WCC (x10^9^/L) (4.0-11.0)	17.2 (H)	32.6 (H)	-	23.4 (H)	-	-
Platelets (x10^9^/L) (150-450)	563 (H)	612 (H)	-	350	-	-
Neutrophils (x10^9^/L) (2.0-8.0)	14.4 (H)	28.9 (H)	-	19.9 (H)	-	-
CRP (mg/L) (0-5)	12 (H)	-	-	-	-	-
INR (0.8-1.2)	-	1.1	-	1.1	-	-
APTT (seconds) (22-32)	-	24	-	24	-	-
Fibrinogen level (g/L) (1.5-4.0)	-	6.2 (H)	-	4.0	-	-

**Table 2:** Summary timeline of clinical events from presentation to discharge. ENDOVAC, endoscopic vacuum-assisted closure; MTP, massive transfusion protocol; CT, computed tomography

Timepoint	Event	Key findings/actions
Day 0 (Presentation)	ED presentation	Acute LUQ pain, hemodynamically stable; Hb 152 g/L, lactate 2.7 mmol/L; CT: large perisplenic hematoma
Day 0 (Deterioration)	Hemodynamic compromise	BP 89/55 mmHg, Hb 84 g/L, lactate 8.2 mmol/L; MTP activated
Day 0 (Procedure)	Splenic artery embolization	Embolization of the inferior pole branches of the splenic artery; transferred to the ICU.
Day 2	Repeat CT	Persistent perisplenic hematoma, suspected pancreatic body/tail infarction, large-volume hemoperitoneum
Day 3	Emergency laparotomy + total splenectomy	Shattered spleen; hemoperitoneum evacuated; specimen sent for histopathology
Day 6	Gastric perforation identified	CT: 2 cm perforation at the greater curve of the stomach; subdiaphragmatic abscess in the splenectomy bed
Day 6+	Endoscopy + ENDOVAC	Ulcerative esophagitis with perforation confirmed; ENDOVAC performed
Subsequent days	Conservative management	Gastrocutaneous fistula managed with antibiotics and abdominal drainage; gradual clinical improvement.
Discharge	Stable discharge	Asplenia vaccination and antimicrobial prophylaxis protocols initiated per guidelines

While awaiting further risk stratification in the emergency department, the patient became acutely hypotensive (blood pressure 89/55 mmHg) responsive to intravenous fluids with a precipitous drop in hemoglobin from 152 g/L to 84 g/L and a rise in lactate to 8.2 mmol/L. Massive transfusion protocol (MTP) was activated. Following multidisciplinary discussion, an emergency decision was made to pursue splenic artery embolization. The interventional radiology procedure involved uncomplicated embolization of the inferior pole branches of the splenic artery, after which the patient was transferred to the intensive care unit for ongoing monitoring.

**Figure 2:**
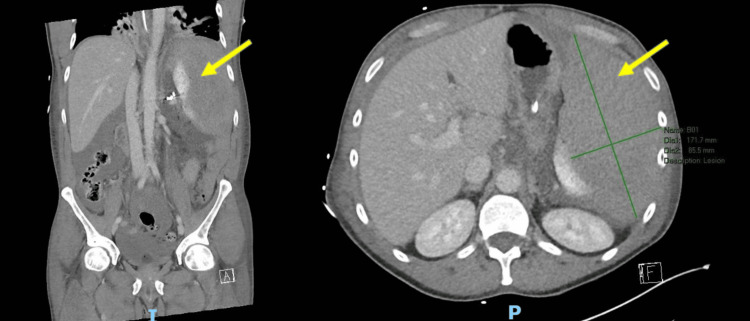
CT abdomen and pelvis demonstrating a persistent splenic hematoma (yellow arrow), suspected infarction of the pancreatic body and tail, and large-volume ascites consistent with hemoperitoneum. Coronal section on the left; axial section on the right. CT, computed tomography

**Figure 3:**
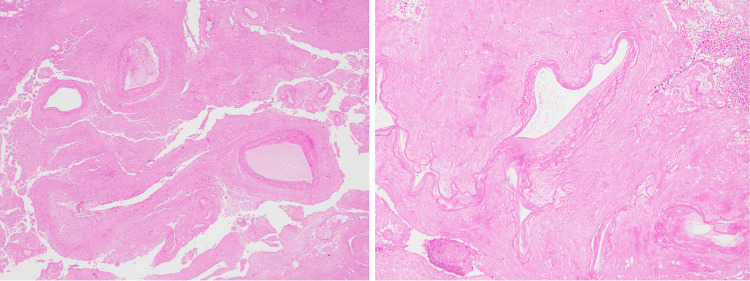
A cluster of mostly necrotic, thick-walled vasculature with a prominent internal elastic lamina and a thickened intimal layer, H&E ×40 (left). A necrotic, irregular vessel wall with a prominent internal elastic lamina and surrounding necrotic tissue, H&E ×100 (right). H&E, hematoxylin and eosin

**Figure 4:**
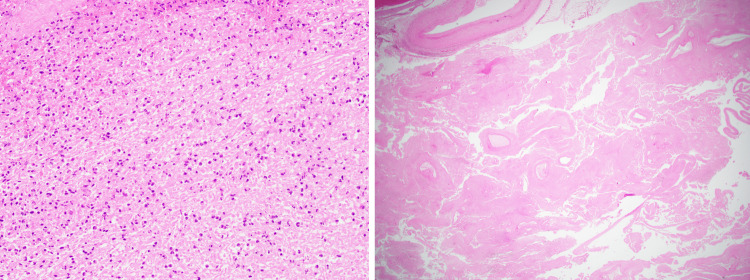
Low-power view of extensive necrosis in the spleen, H&E ×40 (left). Low-power view showing multiple prominent thick-walled vascular channels within the necrotic spleen, H&E ×12.5 (right). H&E, hematoxylin and eosin

Repeat CT performed on Day 2 post-embolization identified persistent perisplenic hematoma, suspected infarction of the pancreatic body and tail, and large-volume ascites consistent with hemoperitoneum (Figure 2). Given these findings and ongoing abdominal pain, the patient returned to the operating theatre on Day 3 for exploratory laparotomy. Intraoperative findings revealed a shattered spleen with extensive hemoperitoneum. Evacuation of the hemoperitoneum was performed, and the remaining splenic parenchyma was removed via total splenectomy. The resected specimens were sent for histopathological analysis (macroscopic and microscopic descriptions below; representative slides in Figure 3-4).

Macroscopic description

Spleen

A disrupted piece of spleen measuring 73 × 37 × 15 mm and weighing 12 g was received. The capsule was smooth, shiny, and pale. The cut surface was soft and partially hemorrhagic brown. No lesions were identified.

Microscopic description

The sections of spleen demonstrated a large area of infarction with tissue necrosis and stromal hemorrhage. Small areas of viable splenic tissue were present. The degenerated area showed outlines of numerous closely opposed vascular channels with elastic lamina, raising the possibility of a vascular malformation with a thick-walled vascular component. There was no evidence of malignancy. 

**Figure 5:**
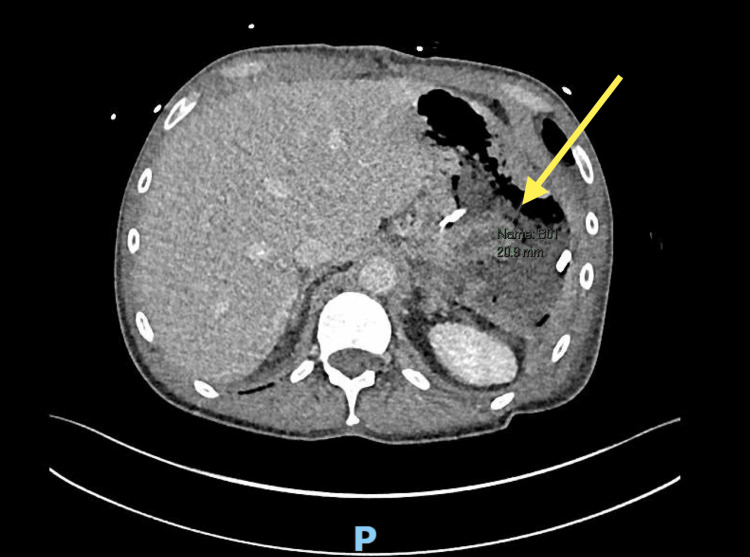
CT abdomen demonstrating a large 21 mm defect in the greater curvature of the stomach (yellow arrow).

The patient's immediate postoperative course was complicated by a gastric perforation identified on Day 6, when repeat CT demonstrated a 2 cm perforation in the greater curve of the stomach (Figure 5). Endoscopy confirmed ulcerative esophagitis with perforation and an adjoining subdiaphragmatic abscess cavity in the splenectomy bed. Endoscopic vacuum-assisted closure (ENDOVAC) was performed. Subsequent imaging demonstrated persistent but improving fistulous communication between the stomach and abdominal drain, which was managed conservatively with antibiotic coverage for the intra-abdominal collection. The remainder of his hospital stay was largely unremarkable, and he was eventually discharged in stable condition.

## Discussion

The pathophysiology of ASR is complex and multifactorial. As outlined in the Introduction, the overwhelming majority of cases occur in pathologically abnormal spleens, with hematological malignancy, viral infection, and inflammatory conditions representing the most commonly reported etiologies. Exogenous hormonal and pharmacological agents represent an emerging and underappreciated etiological category that warrants careful consideration when standard causes have been excluded [7,8].

Our patient’s recent use of RAD-140 and MK-677 raises the question of a possible contribution from these performance-enhancing compounds, building on the established association between traditional anabolic-androgenic steroids and peliosis hepatis and lienis described in the Introduction. Without any prior published association between these compounds and splenic injury, this case is necessarily hypothesis-generating rather than confirmatory. Selective androgen receptor modulators are relatively novel compounds marketed to increase muscle mass with purportedly fewer adverse effects than traditional anabolic steroids; however, as noted, their adverse effect profile is increasingly characterized and includes hepatotoxicity and prothrombotic risk. MK-677, a growth hormone secretagogue, elevates circulating IGF-1 levels via ghrelin receptor agonism and has been used off-label for muscle-building purposes. While both compounds remain unapproved for clinical use, their pharmacological activity on androgen receptor and IGF-1 signaling pathways means extrapolation of known steroid-associated risks may be worth exploring in future research.

Two plausible but speculative mechanistic pathways warrant consideration. First, MK-677-mediated elevation of IGF-1 may promote a prothrombotic or hypercoagulable state, potentially facilitating splenic infarction in the context of an underlying vascular lesion. IGF-1 has recognized effects on platelet aggregation, endothelial function, and coagulation factor activity. Second, sustained IGF-1 elevation may promote vascular remodeling or angiogenesis, potentially compromising the structural integrity of pre-existing vascular malformations and predisposing them to rupture. The histopathological findings in our case - large areas of splenic infarction with features raising the possibility of an underlying vascular malformation - are consistent with either mechanism; however, this remains speculative, and a comprehensive literature search revealed no reported cases of spontaneous splenic rupture directly attributed to SARM or GHS use as of this writing.

The histopathological description of closely opposed vasculature with elastic laminae raises the possibility of a pre-existing developmental or genetic vascular malformation - a lesion that may have been entirely independent of any exogenous agent. Splenic vascular malformations, including cavernous hemangiomas and arteriovenous malformations, are well recognized to remain clinically silent until precipitated by physiological stress, hemodynamic changes, or superimposed infarction. In this context, the performance-enhancing compounds - and indeed olanzapine - may be better understood as potential precipitating or contributing factors rather than primary causes.

An important and underappreciated confounder in this case is the patient's long-term use of olanzapine (20 mg nocte). Olanzapine, an atypical antipsychotic, has been associated with an increased risk of venous thromboembolism and a prothrombotic tendency, potentially mediated through effects on platelet aggregation, clotting factor activity, and hyperhomocysteinemia. Importantly, the patient had been prescribed olanzapine for a substantially longer duration than the recently initiated performance-enhancing compounds, suggesting it may represent a more sustained prothrombotic risk factor. While a causal role for olanzapine in this case cannot be established, it must be acknowledged as a clinically relevant confounder when interpreting any association between SARM or GHS use and the observed outcome.

Regarding other medication-related etiologies of ASR, anticoagulant use and procedural trauma (e.g., post-colonoscopy rupture) are documented causes; neither was relevant in this patient, who was not on anticoagulation and had no recent procedures.

Clinical presentation of ASR is often nonspecific, and a high index of suspicion is required, particularly in patients without a history of trauma. Physical examination typically reveals an enlarged spleen in addition to left upper quadrant tenderness and signs of peritonism [9]. Blood tests may demonstrate nonspecific findings such as anemia and leucocytosis. It is imaging, therefore, that most definitively establishes the diagnosis. While bedside ultrasound is frequently employed in hemodynamically unstable patients, contrast-enhanced CT scanning remains the gold standard, with significantly higher sensitivity (85.7% versus 57.1%) for detecting hemoperitoneum, splenic hematoma, or parenchymal hypodensity suggestive of infarction [2]. The American Association for the Surgery of Trauma (AAST) grading scale, commonly used for traumatic splenic injuries, can also be applied to ASR cases to guide management decisions [10].

Management of ASR hinges on hemodynamic stability and the presence of underlying splenic pathology. In hemodynamically stable patients without active bleeding, conservative management with close observation may be appropriate. Proximal or selective splenic artery embolization represents an attractive alternative that can arrest bleeding while potentially preserving immune function. However, failure rates for embolization can reach 15% to 30%, particularly when underlying splenic pathology renders the parenchyma friable or when diffuse bleeding or coagulopathy is present [11]. A recent analysis by Rippel found that non-operative management - whether observation alone or with embolization - was less successful in atraumatic cases compared to traumatic splenic injuries, leading to worse outcomes when surgical intervention was delayed [12].

Early conversion to total splenectomy is therefore crucial at the first sign of rebleeding or ongoing hemodynamic instability. In addition to providing definitive hemorrhage control, splenectomy permits complete histopathological examination, which is essential for establishing a definitive etiological diagnosis and - as this case illustrates - may yield unexpected findings such as possible underlying vascular pathology that would not have been identified without surgical resection. However, splenectomy is not without long-term consequences. Asplenic individuals carry a lifelong risk of overwhelming post-splenectomy infection (OPSI) and an increased risk of arteriovenous thrombosis [13]. Therefore, all patients undergoing splenectomy require appropriate vaccination and antimicrobial prophylaxis according to established asplenia protocols.

## Conclusions

Atraumatic splenic rupture is a life-threatening surgical emergency requiring rapid recognition and definitive management. To our knowledge, this represents the first reported case of ASR occurring in temporal association with the use of performance-enhancing compounds - specifically a selective androgen receptor modulator (RAD-140) and a growth hormone secretagogue (MK-677). However, the presence of histopathological features raising the possibility of a pre-existing vascular malformation suggests this structural lesion may have been the primary predisposing factor, with exogenous compounds - and potentially olanzapine - serving as additional contributors at most. The attribution of causality in a single case is speculative, and this report is best interpreted as hypothesis-generating.

Three key clinical take-home messages emerge: (1) ASR should be considered in any patient presenting with acute abdominal pain and left upper quadrant peritonism, even in the absence of trauma; (2) a comprehensive medication and supplement history - including over-the-counter performance-enhancing compounds - is essential in all such presentations; and (3) while splenic artery embolization is a valuable adjunct in hemodynamically stable patients, timely conversion to total splenectomy is critical in those with ongoing instability or failure of non-operative management, and histopathological examination of the resected spleen provides indispensable diagnostic information. Larger case series and pharmacovigilance registries are needed to determine whether a true association exists between SARM or GHS use and splenic complications.
